# Randomized, Double-Blind, Placebo-Controlled Trial of a Throat Spray with Selected Lactobacilli in COVID-19 Outpatients

**DOI:** 10.1128/spectrum.01682-22

**Published:** 2022-09-26

**Authors:** Ilke De Boeck, Eline Cauwenberghs, Irina Spacova, Thies Gehrmann, Tom Eilers, Lize Delanghe, Stijn Wittouck, Peter A. Bron, Tim Henkens, Imane Gamgami, Alix Simons, Ingmar Claes, Joachim Mariën, Kevin K. Ariën, Diana Bakokimi, Katherine Loens, Kevin Jacobs, Margareta Ieven, Patricia Bruijning-Verhagen, Peter Delputte, Samuel Coenen, Veronique Verhoeven, Sarah Lebeer

**Affiliations:** a Research Group Environmental Ecology and Applied Microbiology, Department of Bioscience Engineering, University of Antwerp, Antwerp, Belgium; b Yun NV, Niel, Belgium; c Virology Unit, Department of Biomedical Sciences, Institute of Tropical Medicine, Antwerp, Belgium; d Evolutionary Ecology Group, Department of Biology, University of Antwerp, Antwerp, Belgium; e Department of Biomedical Sciences, University of Antwerp, Antwerp, Belgium; f Laboratory of Microbiology, Parasitology and Hygiene, University of Antwerp, Antwerp, Belgium; g Julius Centre for Health Sciences and Primary Care, Department of Epidemiology, University Medical Centre Utrecht, Utrecht, The Netherlands; h Family Medicine and Population Health (FAMPOP), University of Antwerp, Antwerp, Belgium; i Vaccine and Infectious Disease Institute (VAXINFECTIO), University of Antwerp, Antwerp, Belgium; j Department of Microbiology, University Hospital Antwerp, Edegem, Belgium; University of California, San Diego

**Keywords:** COVID-19, lactobacilli, microbiome, throat spray

## Abstract

Primary care urgently needs treatments for coronavirus disease 2019 (COVID-19) patients because current options are limited, while these patients who do not require hospitalization encompass more than 90% of the people infected with severe acute respiratory syndrome coronavirus 2 (SARS-CoV-2). Here, we evaluated a throat spray containing three *Lactobacillaceae* strains with broad antiviral properties in a randomized, double-blind, placebo-controlled trial. Before the availability of vaccines, 78 eligible COVID-19 patients were randomized to verum (*n* = 41) and placebo (*n* = 37) within 96 h of a positive PCR-based SARS-CoV-2 diagnosis, and a per-protocol analysis was performed. Symptoms and severity were reported daily via an online diary. Combined nose-throat swabs and dried blood spots were collected at regular time points in the study for microbiome, viral load, and antibody analyses. The daily reported symptoms were highly variable, with no added benefit for symptom resolution in the verum group. However, based on 16S V4 amplicon sequencing, the acute symptom score (fever, diarrhea, chills, and muscle pain) was significantly negatively associated with the relative abundance of amplicon sequence variants (ASVs) that included the applied lactobacilli (*P* < 0.05). Furthermore, specific monitoring of these applied lactobacilli strains showed that they were detectable via quantitative PCR (qPCR) analysis in 82% of the patients in the verum group. At the end of the trial, a trend toward lower test positivity for SARS-CoV-2 was observed for the verum group (2/30; 6.7% positive) than for the placebo group (7/27; 26% positive) (*P* = 0.07). These data indicate that the throat spray with selected antiviral lactobacilli could have the potential to reduce nasopharyngeal viral loads and acute symptoms but should be applied earlier in the viral infection process and substantiated in larger trials.

**IMPORTANCE** Viral respiratory tract infections result in significant health and economic burdens, as highlighted by the COVID-19 pandemic. Primary care patients represent 90% of those infected with SARS-CoV-2, yet their treatment options are limited to analgesics and antiphlogistics, and few broadly acting antiviral strategies are available. Microbiome or probiotic therapy is a promising emerging treatment option because it is based on the multifactorial action of beneficial bacteria against respiratory viral disease. In this study, an innovative topical throat spray with select beneficial lactobacilli was administered to primary COVID-19 patients. A remote study setup (reducing the burden on hospitals and general practitioners) was successfully implemented using online questionnaires and longitudinal self-sampling. Our results point toward the potential mechanisms of action associated with spray administration at the levels of viral loads and microbiome modulation in the upper respiratory tract and pave the way for future clinical applications of beneficial bacteria against viral diseases.

## INTRODUCTION

During the coronavirus disease 2019 (COVID-19) pandemic, most research and clinical trials on treatment options have been conducted with hospitalized patients. This especially applies to intervention studies, which are routinely executed in a hospital setting with critically ill patients. However, only 10 to 20% of COVID-19 patients need medical care in hospitals (1). While these numbers vary depending on the dominating severe acute respiratory syndrome coronavirus 2 (SARS-CoV-2) variant, this means that the majority of COVID-19 patients have mild-to-moderate symptoms, are not hospitalized, and depend only on treatments such as antiphlogistics and analgesics (1, 2). Nevertheless, these milder cases exert a significant burden on health care professionals in primary care (2, 3). In addition, asymptomatic and presymptomatic transmission are the main drivers of transmission to others (4). Respiratory viral infections can have severe health consequences due to imbalanced immune activation and bacterial coinfections associated with airway tissue disruption and severe inflammation (5). This clearly shows the urgent need for more treatment and/or prevention options in COVID-19 outpatients, which can improve different aspects of the disease: symptom relief, transmission reduction, and decreased hospitalizations. Most focus until now has been on the development of vaccines, which appear to prevent severe disease and stimulate antibody and cellular immunity (6–8). Recently, several randomized, controlled trials have also been conducted with outpatients, focusing on monoclonal antibodies (9–11) and fluvoxamine (12), which showed a reduced risk of hospitalization and/or reduced viral loads. However, their abilities to prevent infection, block mucosal viral replication in the upper airways, and decrease the transmission of different virus variants in a cost-effective way appear to be limited (13, 14).

Microbiome or probiotic therapy is an emerging alternative treatment option for respiratory viral diseases because it is based on the multifactorial action of selected beneficial bacteria in or on the airways (15). While the oral administration of such microbiome therapeutics or probiotics remains the most common (16), this route relies solely on systemic effects to ameliorate respiratory infections. During the current COVID-19 pandemic, the oral administration of probiotics using formulations delivering the bacteria directly in the gut, such as powder (Sivomixx [17]) and capsules (18), has already been explored. Alternatively, the topical application of rationally selected probiotics in the airways might offer several advantages (19); as such, local administration could lead to the direct blocking or inhibition of respiratory viruses (20) and direct immune modulation at the site of infection and inflammation (21, 22). Indeed, the probiotic definition is not limited to the gut (23). We have recently developed an antiviral throat spray with three *Lactobacillaceae* strains that were selected based on their safety, their *in vitro* multifactorial modes of action on key aspects of viral infection and disease (including interferon activation and blocking of upper respiratory tract [URT] viral infection), and their ability to thrive in the human respiratory tracts of healthy volunteers (24). Yet microbiome therapy in patients with live bacteria has several challenges, such as formulations, regulation, and selection of the target patient population.

Here, we evaluated the clinical potential of this multispecies probiotic throat spray with Lacticaseibacillus casei AMBR2, Lacticaseibacillus rhamnosus GG, and Lactiplantibacillus plantarum WCFS1 against COVID-19 in a randomized, double-blind, placebo-controlled trial in unvaccinated COVID-19 outpatients exhibiting mild-to-moderate symptoms. The formulation with L. casei AMBR2, L. rhamnosus GG, and L. plantarum WCFS1 was developed based on the safety, applicability, and experimentally observed functional characteristics of several strains screened (immunostimulation, direct antiviral action, and epithelial barrier maintenance), as described in detail previously (24). In this study, we monitored the impact of these previously selected strains in the spray on symptom severity, time to improvement, viral loads, anti-SARS-CoV-2 antibodies, and the respiratory microbiome in an out-of-hospital setting. This trial was designed based on self-collected samples of combined nose-throat swabs, fingerprick blood samples, and reporting of symptoms and severity via an online diary and was run before the broad vaccine campaign was launched in Belgium.

## RESULTS

### Setup of a placebo-controlled intervention trial in mild-to-moderate COVID-19 patients.

**FIG 1:**
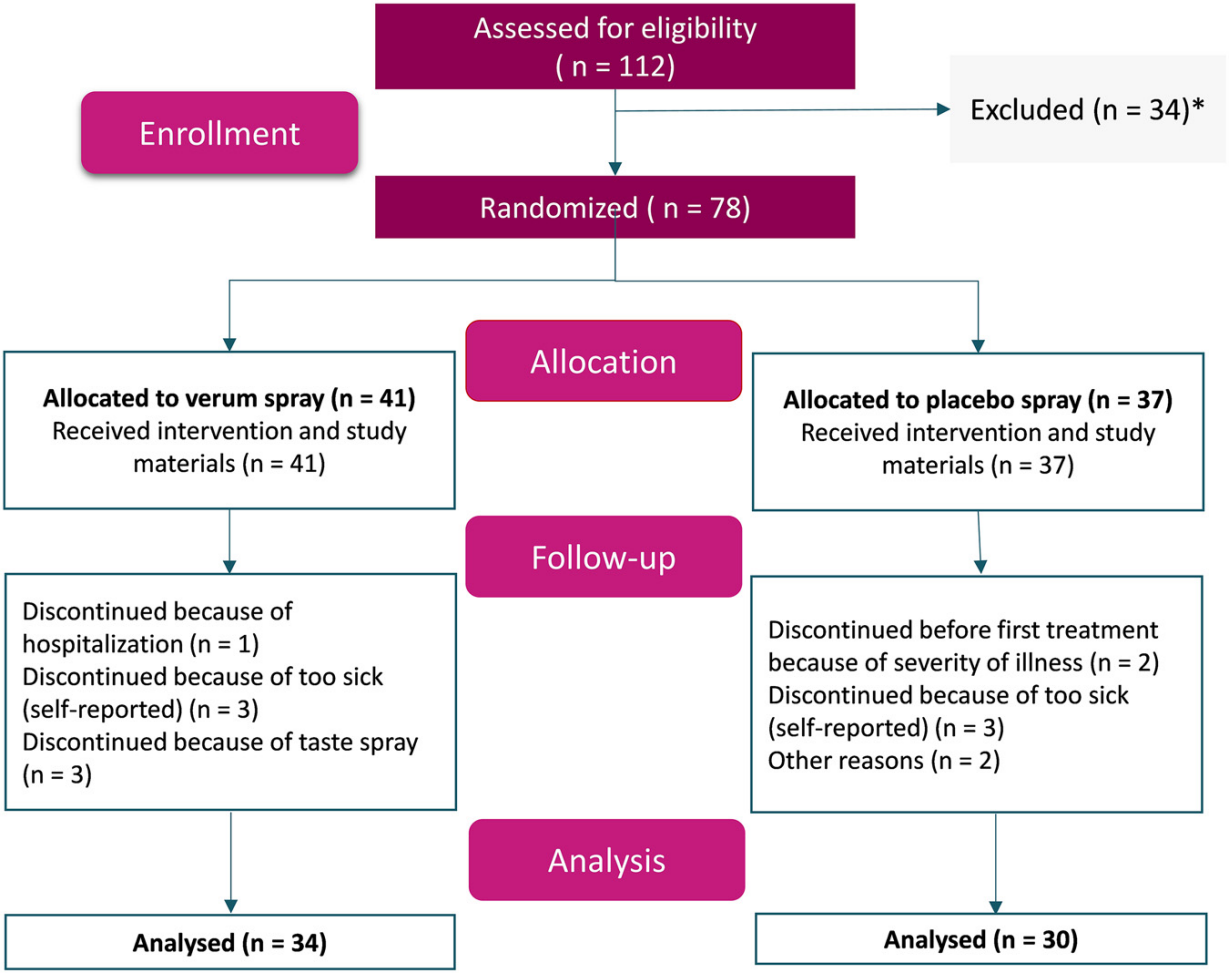
Consolidated Standards of Reporting Trials (CONSORT) flowchart for patient recruitment and enrollment. *, excluded for not meeting the inclusion criteria (mostly because the time from a positive PCR test was >96 h, often when people contacted the study coordinator in response to the press release message) or declining to participate after the first contact with the study coordinator. Of note, for the analysis of SARS-CoV-2 loads by RT-qPCR, 4 participants in the verum group and 3 participants in the placebo group already had a negative PCR test at *T*_1_ of our study. Hence, for this analysis, 30 participants and 27 participants were analyzed for verum and placebo, respectively.

Seventy-eight eligible unvaccinated patients were randomized, of which 41 were allocated to the verum spray and 37 were allocated to the placebo spray. Figure 1 depicts patient recruitment and enrollment. Fourteen participants dropped out during the trial (7 in the verum group and 7 in the placebo group) (reasons are shown in Fig. 1), and a per-protocol analysis of the remaining participants who provided samples at all time points was conducted. Patient demographics, reported symptoms at enrollment, and the time between a positive PCR test and the start of the intervention are shown in Table 1.

**TABLE 1:** Patient demographics and baseline characteristics by treatment group^*a*^

Characteristic	Value for group	
	Verum (*n* = 33)	Placebo (*n* = 27)
Mean age (yrs) ± SD	42 ± 12	43 ± 12
No. (%) of female patients	21 (62)	19 (63)
Mean BMI (kg/m^2^) ± SD	26.6 ± 4.8	26.1 ± 5.5
No. (%) of obese patients (BMI > 30)	4 (12)	5 (19)
No. (%) of smokers	4 (12)	5 (19)
No. (%) of patients with employment in:		
Patient care	1 (3)	1 (4)
Child care	2 (6)	1 (4)
Teaching	7 (21)	5 (19)
Other	23 (70)	20 (74)
No. (%) of patients with inhalation allergy	15 (45)	7 (26)
No. (%) of patients with lung disease (asthma, COPD)	3 (9)	4 (15)
No. (%) of patients with cardiac disease	2 (6)	0 (0)
No. (%) of patients with immune disorder	1 (3)	1 (4)
No. (%) of patients with diabetes	1 (3)	1 (4)
No. (%) of patients with hypertension	3 (9)	4 (15)
Median no. of days from positive PCR test (range)	1 (1–3)	1 (0–4)
Median no. of days since onset of symptoms (range)	3 (1–18)	2 (1–11)
No. (%) of patients with fever	5 (15)	7 (26)
No. (%) of patients with cough	24 (73)	17 (63)
No. (%) of patients with sore throat	13 (39)	9 (33)
No. (%) of patients with runny/blocked nose	20 (61)	22 (81)
No. (%) of patients with shortness of breath	7 (21)	7 (26)
No. (%) of patients with headache	21 (64)	18 (67)
No. (%) of patients with loss of smell and taste	8 (24)	5 (19)
No. (%) of patients with muscle pain	17 (52)	15 (56)
No. (%) of patients with chills	13 (39)	7 (26)
No. (%) of patients with fatigue	25 (76)	20 (74)
No. (%) of patients with diarrhea	2 (6)	2 (7)
No. (%) of patients positive for SARS-CoV-2 IgG at study start	2 (6)	3 (12)^*b*^
No. (%) of patients with comedication at study start	20 (61)	21 (78)
Mean reported no. of days of usage of comedication	11.0 ± 8.2	8.8 ± 7.5

a Due to missing values for 4 participants who did not complete or fill out the intake survey, this table is based on data from 60 participants. BMI, body mass index; COPD, chronic obstructive pulmonary disease.

b For 1 participant, we did not have antibody data, so only 26 participants were included for this result.

The sprays were overall well tolerated, although several participants in both study groups reported an unpleasant taste (mainly in the verum group) or texture (verum and placebo) of the spray. For the online diaries, compliance was high: the median number of completed diary entries was 20/21 days. The compliance rates for self-sampling were 80.5% (509/632) for the combined nose-throat swabs and 83.5% (132/158) for the fingerprick blood samples.

### Monitoring of symptoms in primary care patients and impacts of the throat spray on symptom severity and time to improvement.

Symptoms at the start of the study are shown in Table 1. Cough (68%), runny/blocked nose (70%), headache (65%), and fatigue (75%) were the symptoms most frequently reported. The average total symptom scores at the start of the study were 13.4 ± 8.6 in the verum group and 15.2 ± 9.3 in the placebo group (the difference was not significant) (see Table S1 in the supplemental material).

**FIG 2:**
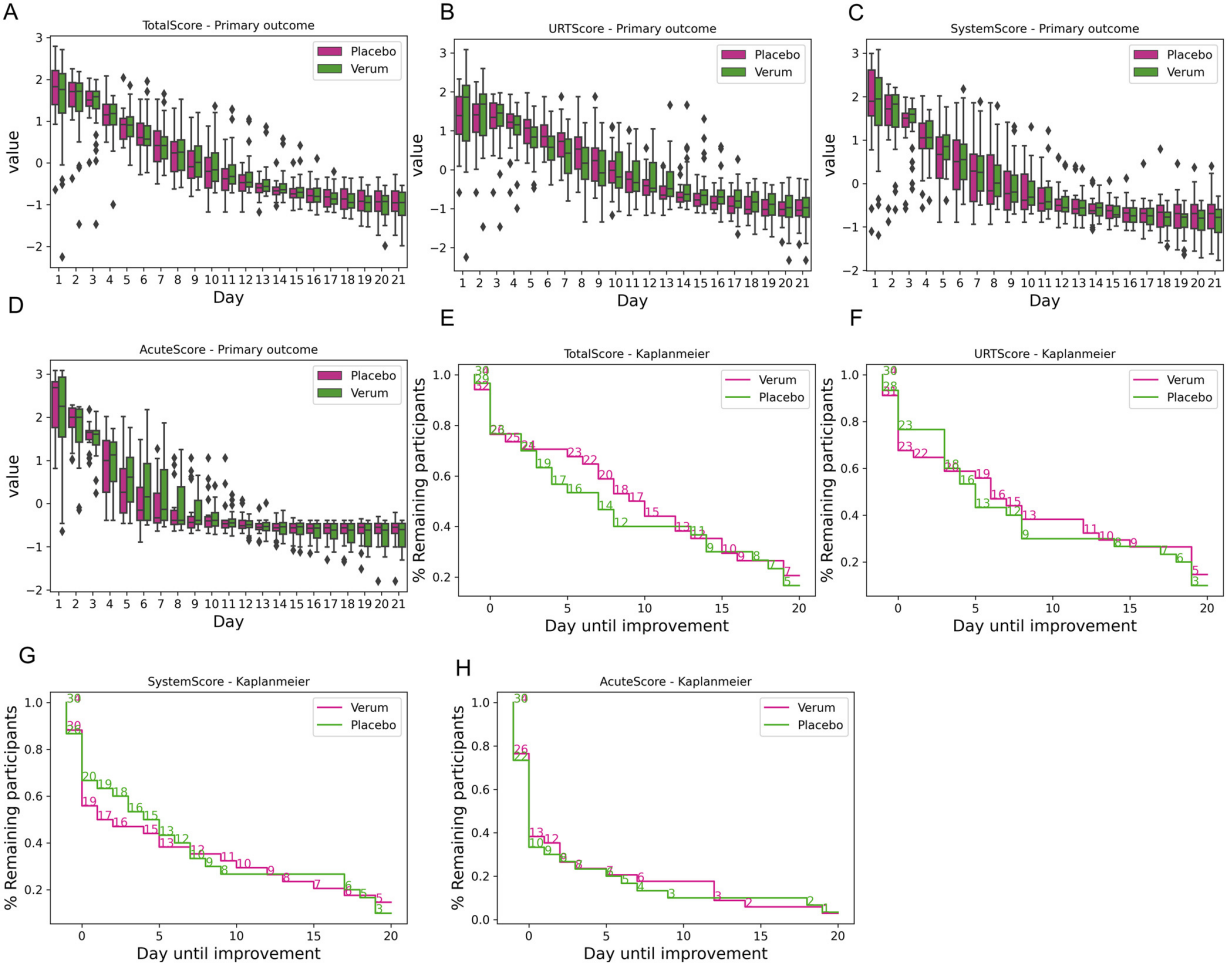
Symptom severity and time to improvement. (A to D) The severity of the reported symptoms was evaluated based on different scoring systems: the total score (A), URT score (B), system score (C), and acute score (D). Results are shown as standardized scores (z scores) to adjust for the highly subjective self-evaluation. (E to H) The time to improvement was also evaluated for the 4 scoring systems between the study groups. Survival analysis showed no significant differences for all tested scores between placebo and verum (*P* > 0.1).

The severity of the symptoms was evaluated for both treatment groups during the study via the distribution of the different severity scores (total, URT, acute, and symptom scores) at every day (Table S1). The same tendencies for the verum and placebo groups were observed, with no significant differences (Fig. 2A to D). Independent of treatment, raw symptom scores showed high inter- and intraindividual fluctuation patterns (Fig. S2), and scores were therefore propagated, smoothened, and standardized (Fig. S3). The time to improvement was not significantly different between treatment groups: the log odds (Cox regression) were 0.125 ± 0.3, −0.003 ± 0.3, 0.111 ± 0.3, and 0.58 ± 0.3 for the total, system, URT, and acute scores, respectively (Fig. 2E to H). Over the entire study population, 59% of the individuals (independent of treatment) still experienced symptoms after 21 days. At this 3-week time point, 5% reported acute symptoms, 39% reported systemic symptoms, and 41% reported URT symptoms.

### Impact of the microbiome spray on test positivity for SARS-CoV-2 and relationship to symptoms.

**FIG 3:**
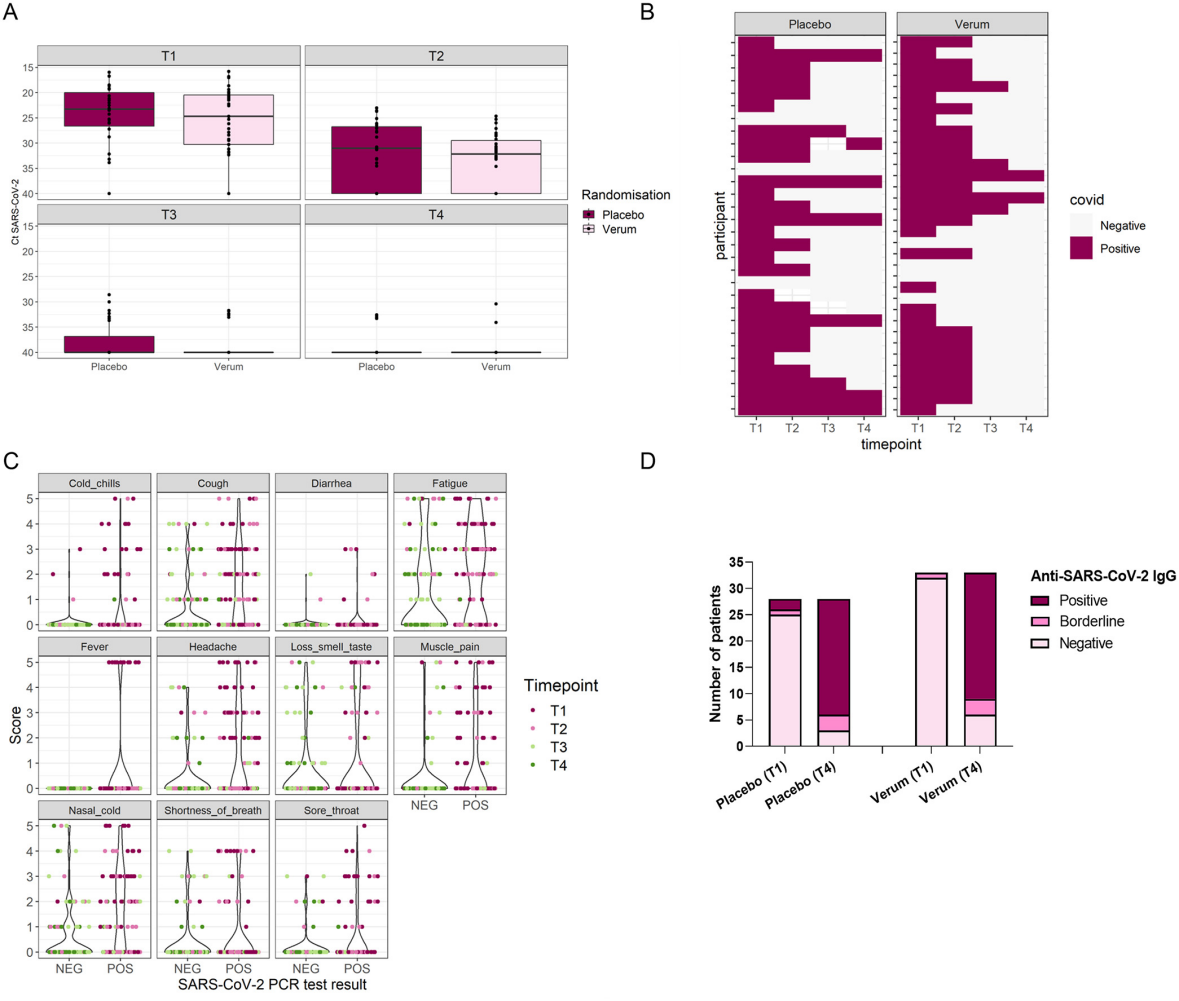
Viral loads in combined nose-throat swabs. (A) SARS-CoV-2 loads in combined nose-throat swabs determined via PCR at the start (*T*_1_), after 1 week (*T*_2_), after 2 weeks (*T*_3_), and after 3 weeks/at the end of the study (*T*_4_). Results are shown as *C_T_* values. (B) Heat map showing the presence or absence of SARS-CoV-2 based on a positive PCR test per participant. (C) Relationship between self-reported symptoms and SARS-CoV-2 loads. (D) Presence of anti-SARS-CoV-2 IgG antibodies in the blood of COVID-19 patients (COV) at the start (*T*_1_) and end (*T*_4_) of the study, comprising 3 weeks in between. Data for the verum and placebo treatment groups are depicted as the numbers of participants positive, borderline, or negative for anti-SARS-CoV-2 IgG as part of the total number of participants per treatment group per time point. Only participants with blood samples available at both *T*_1_ and *T*_4_ were included in this analysis.

At the start of the trial, 4/34 participants in the verum group and 3/30 participants in the placebo group had a negative reverse transcription-quantitative PCR (RT-qPCR) result despite testing positive less than 96 h earlier. After 1 week, 73% of the participants in the verum group and 77% in the placebo group tested positive (*P* = 1 by Fisher’s exact test), while after 2 weeks, these values were 17% and 32%, respectively (*P* = 0.22 by Fisher’s exact test). At the end of the trial, 2/30 (6.7%) patients in the verum group and 7/27 (26%) patients in the placebo group still tested positive (*P* = 0.07 by Fisher’s exact test) (Fig. 3A and B). Independent of the intervention, all symptoms had a strong correlation with SARS-CoV-2 positivity (Table S2), although several symptoms, such as cough, nasal symptoms, and fatigue, were still reported upon a negative PCR result (Fig. 3C).

Analysis of self-collected fingerprick blood samples to assess antibody responses (see reference 25) showed that at the start of the study, only 4/61 of the enrolled unvaccinated COVID-19 patients were positive or borderline positive for anti-SARS-CoV-2 IgG based on antibody reactivity against the receptor-binding domain (RBD), nucleocapsid protein (NCP), and spike proteins (S1S2) of SARS-CoV-2 (26) (Fig. 3D). After 3 weeks, 51/61 patients were positive or borderline positive for anti-SARS-CoV-2 IgG, without significant differences between the placebo and verum groups (*P* = 0.71 by a chi-square test).

### Impact of SARS-CoV-2 infection and lactobacilli treatment on the upper airway microbiome.

**FIG 4:**
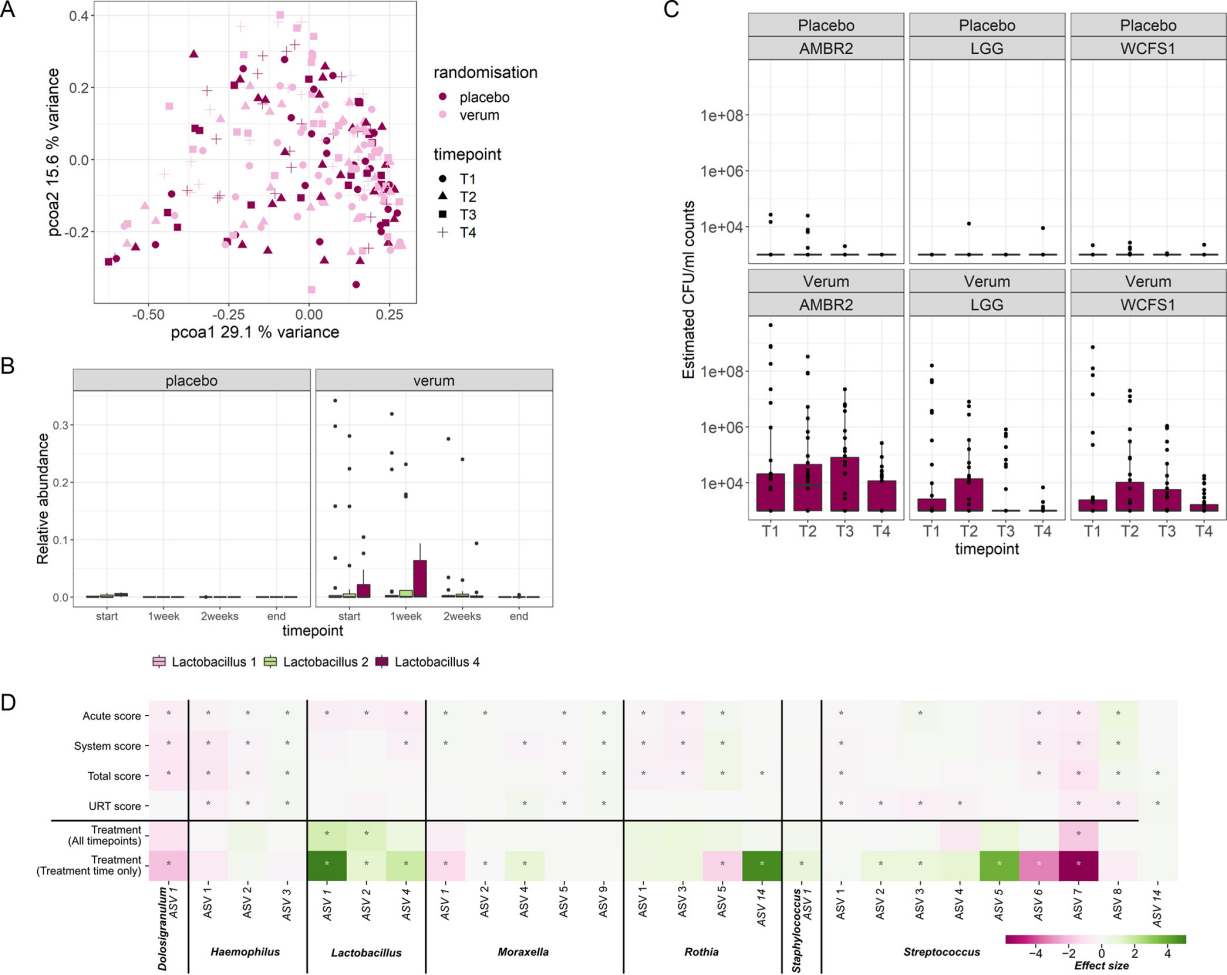
Microbial community composition in the airways (A), detection of the administered *Lactobacillaceae* strains (B and C), and association of treatment and symptom scores with bacterial taxa (D). (A) PCoA was used to visualize the microbiome composition in combined nose-throat swabs for each treatment group and at different time points. *T*_1_, start of the trial; *T*_2_, after 1 week; *T*_3_, after 2 weeks; *T*_4_, end of the trial. (B) Relative abundances of *L. casei* ASV1, *L. plantarum* ASV2, and L. rhamnosus ASV3 between the placebo and verum groups. See also Table S2 in the supplemental material for the mean relative abundances for all ASVs at different time points and statistics. (C) qPCR with species-specific primers for L. rhamnosus GG, *L. casei* AMBR2, and *L. plantarum* WCFS1 was used to estimate the counts of CFU per milliliter. Based on the standard curve, the detection limit was estimated to be 10^3^ CFU/mL. (D) Association of treatment and symptom scores with bacterial taxa across all time points in the study. For treatment, the association was also evaluated for treatment time only.

Principal-coordinate analysis (PCoA) showed no major shifts in the overall nose/throat microbial composition for the time points of viral infection or for the microbiome treatment (Fig. 4A). However, specific effects on the abundances of certain taxa were observed. When focusing on the abundances of the amplicon sequence variants (ASVs) containing the *Lactobacillaceae* strains administered with the throat spray, significant differences were observed between the verum and placebo groups at different time points, with mean relative abundances for the *L. casei* ASV, *L. plantarum* ASV, and L. rhamnosus ASV in the verum group of 1.6%, 1.3%, and 0.5%, respectively, over the entire study (Fig. 4B; Table S3). In the placebo group, these numbers were below 0.01% for all three ASVs (Table S3). The prevalences (presence) based on MiSeq data were 38.6%, 28%, and 13.4% for *L. casei* ASV1, *L. plantarum* ASV2, and L. rhamnosus ASV4, respectively, while the prevalences were 10.5%, 7%, and 2% in the placebo group, respectively, showing that taxa related to the applied lactobacilli were also endogenously present but at low numbers. Therefore, the presence and estimated numbers of the specifically applied *Lactobacillaceae* strains were also confirmed via qPCR, with a clear difference between verum and placebo, with estimated counts of CFU per milliliter of the three strains in the ranges of 10^8^ CFU/mL for *L. casei* AMBR2, 10^7^ CFU/mL for *L. plantarum* WCFS1, and 10^6^ CFU/mL for L. rhamnosus GG, in line with the concentrations at which they were added to the throat spray (and detected in 82% of the study population), whereas these numbers were 100 to 10,000 times lower for placebo (Fig. 4C; Table S4).

Next, we associated the relative abundances of a selection of ASVs belonging to important airway genera (*Rothia*, *Dolosigranulum*, Streptococcus, Staphylococcus, Haemophilus, *Moraxella*, and *Lactobacillus*) (Table S5) with treatment, severity scores, and viral loads across all time points and made a further selection based on the ASVs that showed the highest effect sizes (Fig. 4D). *L. casei* ASV (effect size of 5.12), *L. plantarum* ASV2 (effect size of 1.15), and L. rhamnosus ASV4 (effect size of 1.79) showed strong significant enrichment (*P* < 0.05) in the verum group (Fig. 4D). In addition to these deliberately added lactobacilli, other ASVs were significantly (*P* < 0.05) positively associated with the verum group, including *Moraxella* ASV4 (M. lacunata) (effect size of 0.95), *Rothia* ASV14 (R. amarae) (effect size of 4.86), and several commensal Streptococcus ASVs (S. thermophilus, S. rubneri, and S. sanguinis, among others). On the other hand, significant (*P* < 0.05) negative associations with treatment were observed, with the strongest effects being observed for *Dolosigranulum* ASV1 (D. pigrum) (effect size of −1.99), Streptococcus ASV7 (S. gordonii) (effect size of −5.8), and Streptococcus ASV6 (S. crispatus, S. oligofermentans, and S. sinensis) (effect size of −3.3).

Finally, significant (*P* < 0.05) positive and negative associations between the symptom scores and specific taxa, with moderate effect sizes, were found (Fig. 4D). Of interest, a significant negative association was found for the ASVs corresponding to the applied lactobacilli and the acute symptom score, indicating that the application of these lactobacilli could result in less acute symptoms (Table S5). *Dolosigranulum* ASV1 (effect size of −0.53), another lactic acid bacterium, had negative associations with the acute symptom severity score and even with the total score. Conversely, Haemophilus ASV3 (H. aegyptius) was positively associated with the different symptom scores. The viral load did not have significant associations with any specific taxa (*P* > 0.05).

## DISCUSSION

In this study, we evaluated the use of a specifically formulated throat spray with three selected members of the *Lactobacillaceae* in a placebo-controlled, remote self-sampling study in unvaccinated COVID-19 outpatients. Detailed microbiome and qPCR analyses showed the detection of the applied strains in the verum group in 82% of the participants on average based on qPCR, with estimated concentrations of between 10^6^ and 10^8^ CFU/mL. Analysis of the self-reported symptoms showed patient-dependent disease progression with high intra- and interindividual variations and no significant effects of the intervention on the primary outcome in this rather small study population. However, a trend toward faster-decreasing positivity for the presence of the virus was observed in the verum group compared to the placebo group, with 6.7% and 26% of the participants remaining positive after 3 weeks, respectively, based on RT-qPCR testing (*P* = 0.07). This remains to be substantiated in follow-up studies. Yet this is of interest to combine with the present COVID-19 vaccines that induce immunity but appear to be unable to block viral infection and transmission (13, 14).

The first key finding of the study was the personal fluctuations in COVID-19 symptoms, which we could document thanks to online questionnaires that were developed within the Rapid European COVID-19 Emergency Response (RECOVER) research project (25). Another recent study with daily monitoring of symptoms for 2 weeks also reported that the natural course of COVID-19 is highly patient-dependent and variable (27). This fluctuating disease pattern and the subjective self-evaluation of the symptom scores also complicate studies on treatments for COVID-19 symptoms. For instance, no effects of oral azithromycin in outpatients on the absence of self-reported symptoms after 14 days as the primary outcome were observed in a study with 263 patients (28). This was confirmed in the large, open-label, multiarm PRINCIPLE trial for the azithromycin group (*n* = 2,265) (29). A larger trial with inhaled budesonide showed positive effects on at-risk COVID-19 outpatients aged 50 to 65 years (*n* = 4,700), with a benefit in the time to self-reported recovery of 2.9 days for patients in the budesonide group compared to the usual-care group (30). Probiotic trials with COVID-19 outpatients are scarce compared to those for drug interventions, but two recent studies report some symptom improvement. The intranasal administration of Lactococcus lactis W136 in 23 patients was associated with less fatigue in the verum group than in the placebo group on day 7 (*P* = 0.02). In addition, patients in the verum group had a reduced loss of sense of smell on day 9 (*P* = 0.03) and reduced shortness of breath on day 8 (*P* = 0.02) and day 12 (*P* = 0.04) compared to the placebo group (31). However, it should be noted that the authors of that study did not report any correction for multiple testing, and the study is also not yet peer reviewed. In another, larger trial (*n* = 300) with an oral probiotic mixture of *L. plantarum* strains and Pediococcus acidilactici KABP021, patients in the verum group reported fewer days of fever, cough, headache, body aches, and shortness of breath (18).

The second major finding of our work is the lower number of virus-positive test results at day 21, in line with the selected antiviral mode of action *in vitro*, where we showed that the selected lactobacilli could induce interferon regulatory pathways and reduce the cytopathic effects of coronaviruses and related respiratory viruses in cellular models (24). Due to the delay between testing and inclusion, most participants started within 2 to 5 days after the onset of their first symptoms, when the viral loads were the highest. Moreover, we also observed that most patients still experienced symptoms at the end of monitoring (5% still reported acute symptoms, 39% reported systemic symptoms, and 41% reported URT symptoms), so follow-up work with any potential therapeutic or nutritional interventions in outpatients would preferably extend the follow-up period for the patients to evaluate long-COVID effects. The need to start early enough in the viral infection process has also been observed in other trials with antivirals for respiratory infections (32, 33). For local applications of live probiotics or microbiome therapeutics (15), compared to oral applications such as those reported previously (18), it is especially important that the antiviral bacteria are provided early enough in the viral infection process, considering the more local mode of action. Oral probiotics target the gut and systemic immunity to reduce some local viral readouts at later phases (34). For example, in the trial with the oral probiotic *L. plantarum* strains and Pediococcus acidilactici KABP021, a reduction in the nasopharyngeal viral load was observed in the verum group on day 15 and day 30 (*P* < 0.001) (18). Of interest, the same study also demonstrated that certain systemic symptoms such as fever, body aches, or shortness of breath already improved on days 2 to 3 compared to the placebo group.

Our finding of reduced test positivity for SARS-CoV-2 also suggests some yet-to-be-validated potential to reduce transmission to household members, other high-risk contacts, and also vaccinated individuals since the currently available vaccines have shown a limited capacity to prevent transmission (14, 35). In a prospective cohort study conducted in The Netherlands and Belgium between April and December 2020 (alpha variant), it was shown that secondary transmission within households occurred in 44.4% of the households, mostly very early after the index patient was positive (25). Moreover, our data also suggest that it could be useful for future studies to stratify potential responders and nonresponders based on the microbiome. For example, patients with high relative abundances of the lactic acid bacterium *Dolosigranulum* could be excluded because of the negative association with treatment found here. However, this is currently not standard in clinical trials with microbiome therapeutics or probiotics. Moreover, an alternative formulation such as a nasal spray instead of the throat spray used here might be more favorable since SARS-CoV-2 receptors are highly expressed in the nasal epithelium, and the alpha variant that was dominant during the study has been shown to target primarily the nose (36). In addition, based on previous in-house research, the selected lactobacilli have several beneficial modes of action, such as antimicrobial and immunomodulatory properties, as well as barrier-enhancing effects in the nasal epithelium (37–39). However, this will depend on the virus variant that is the most dominant because the current SARS-CoV-2 omicron (B.1.1.529) variant seems to target the throat as the first or main site with high viral loads, and a throat spray thus seems to be the most suitable form.

Since our study was one of the few studies of mild-to-moderate COVID patients outside the hospital and was based mostly on self-sampling, we also generated relevant information irrespective of the treatment evaluated. In addition to the above-mentioned intrapersonal fluctuations and interpersonal variations in disease symptoms observed via our detailed online diaries, we also observed the robust detection of SARS-CoV-2 in self-collected combined nose-throat swabs. This exemplifies that a self-sampling approach for these types of samples is feasible, in line with the previously demonstrated effectiveness of SARS-CoV-2 detection in oropharyngeal swabs collected by self-sampling during early infection compared to other readouts and sample types (40). The collection of samples without the involvement of a third party could reduce exposure, expand testing capacities, and minimize the burden on hospitals and general practitioners during the COVID-19 pandemic (40, 41). Our results indicate the robust detection of IgG against the SARS-CoV-2 RBD, NCP, and S1S2 antigens in self-collected dry blood spot samples, allowing the detection of positive cases without the need for blood collection by health care professionals and potentially facilitating easier serosurveillance among the general population.

Regarding study limitations, major factors were the lack of statistical power and the rather high dropout rate (18%). It would have been of interest to include more participants, but this was not possible toward the end of the study due to fewer COVID-19 cases and a very active vaccination campaign in Belgium (86.4% vaccination rate) (42). The remote self-sampling nature of the study was successful, but it also introduced certain variability between samples and limited the number and types of samples that could be collected to gain more insights into the mechanisms of action of the throat spray. Furthermore, an important limitation of the study was the variation in the time between infection and the start of the intervention with the throat spray, which introduced individual variability in the results. This was out of the control of the study team because it was dependent on the national governmental testing strategy. However, in future research, a preventive setup with spray use before symptoms start would be of great interest for further research, if this can be organized logistically.

## MATERIALS AND METHODS

### Clinical trial design.

A double-blind, placebo-controlled clinical trial was performed with a microbiome throat spray in COVID-19 outpatients who were not yet vaccinated within 96 h after a positive PCR test in government facilities. The trial was conducted from 24 February to 30 April 2021 at the University of Antwerp. During this period, there were 241,629 new cases confirmed, which were dominated by the alpha variant (between 58 and 86%) (Sciensano). Approval was obtained from the Committee of Medical Ethics (UZA/UAntwerpen) (approval number B3002021000018), and the trial was registered at ClinicalTrials.gov (identifier NCT04793997). Informed consent was obtained from all participants prior to inclusion.

### Participants.

COVID-19 outpatients, aged 18 to 65 years, were recruited via general practitioners, local triage centers, and newspaper and radio due to a press release announcing the initiation of this study. Patients were contacted by the study coordinator within 96 h of a positive PCR test to evaluate whether all inclusion and exclusion criteria were met. Upon inclusion, a study package with all materials required for the study was delivered to their place of confinement.

### Randomization and masking.

Randomization occurred in blocks of six patients, with stratification for age and gender, using a randomization list generated with the Sealed Envelope Web service (https://www.sealedenvelope.com/), by the responsible clinician, who was not involved in the outcome assessment and data analysis. All other researchers and doctors involved in the study were blind to patient data. Participants were enrolled and assigned to study groups by the study coordinator at the time of first contact via phone. If several household members participated in the study, randomization was done per household, with treatment being allocated based on the contact person of the household. Verum and placebo products were given the label A or B and provided together with the study package. Participants, investigators, and outcome assessors were blind to the treatment allocation until all analyses were performed, and the study groups were unblinded by the formulation team.

### Intervention.

Verum and placebo sprays were supplied by Yun NV (Niel, Belgium) and were indistinguishable in shape and color. The verum spray consisted of freeze-dried *Lacticaseibacillus casei* AMBR2, *Lactiplantibacillus plantarum* WCFS1, and *Lacticaseibacillus rhamnosus* GG at a ratio of 50% to 33.3% to 16.7%, respectively, in sunflower oil with Aerosil, vitamin D_3_, and vitamin E. Placebo sprays had the same composition but without the bacteria. Aerosil (pure silicon dioxide) is used in pharmaceutical formulations as a thickening agent. Both the verum and placebo sprays could be stored at room temperature. Quality control of the sprays and evaluation of the stability of the strains were performed at Yun NV and included microbial analyses and evaluation of spray characteristics, sedimentation, viscosity, and gelling at different temperatures from 4°C to 25°C. The absence of nonlactobacilli was evaluated on soybean casein digest (CASO) or Sabouraud agar. Both verum and placebo have a total aerobic microbial count of <10^2^ CFU/g and a total count of yeasts and molds of <10^1^ CFU/g, with the absence of pathogenic microorganisms based on pharmacopeial standards for oromucosal use (43, 44).

### Study procedures.

Patients were asked to use the verum spray or placebo for 14 days by spraying two puffs containing approximately 9.5 × 10^8^ CFU of lactobacilli multiple times a day, with 1 week of follow-up, and filled out an online diary via Qualtrics (Qualtrics, Provo, UT, USA) (for a detailed description, see Fig. S1 in the supplemental material). Ten common COVID-19 symptoms were monitored, including cough, sore throat, runny/blocked nose, shortness of breath, headache, muscle pain, chills, fatigue, loss of smell and taste, and fever (≥38°C). Each symptom was given a score of between 0 (no symptoms) and 5 (severe symptoms), according to a scoring system described previously (25), with the exception of fever, which received a binary score. Different symptom summary scores were compared between the verum and placebo groups: the total score (the sum of all reported symptoms), local URT score (the sum of the scores for cough, sore throat, and nasal discomfort), acute score (the sum of the scores for fever, diarrhea, chills, and muscle pain), and system score (the sum of the scores for fever, shortness of breath, muscle pain, chills, fatigue, and diarrhea). The time to improvement was evaluated based on the time point when participants reached their symptomatic tipping point. Therefore, the time interval after which a particular symptom only improved was determined for each participant; i.e., after that time interval, the symptom score was continuously decreasing.

Study compliance was assessed based on responses via the online diary, the self-sampling of combined nose-throat swabs and blood fingerprick samples, and the use of the spray. The latter was evaluated via self-reporting, and the spray bottles were weighed by the study team.

### Sample collection.

Samples were collected via self-sampling, with guided instruction books and videos. The collection of combined nose-throat swabs for microbiome analysis and the determination of SARS-CoV-2 loads were performed every week from the start of the trial for a total of 4 time points (*T*_1_ [at the start of the trial], *T*_2_ [after 1 week], *T*_3_ [after 2 weeks], and *T*_4_ [at the end of the trial]). At *T*_1_, participants were asked to take the sample prior to using the microbiome spray. However, as the microbiome sequencing results indicated high levels of lactobacilli in the verum group even at *T*_1_, we suspect that a subset of participants used the spray before taking the *T*_1_ sample. Therefore, samples at *T*_1_ were used for all analyses except for the statistical analysis of the microbiome data to assess the association of treatment and symptom scores with bacterial taxa. Blood fingerprick samples (dried blood spots) to analyze SARS-CoV-2 IgG antibodies were self-sampled at the start and end of the trial. All samples were stored at −20°C for swabs or 4°C for blood fingerpricks.

### Outcomes.

The primary clinical outcome of this trial was the change in the severity of COVID-19 infection symptoms after using the microbiome spray. The secondary study outcomes included (i) the change in the duration (time to improvement) of COVID-19 infection symptoms after using the microbiome spray, (ii) the change in the absolute level of SARS-CoV-2 particles after using the microbiome spray, (iii) the change in the absolute numbers of specific bacterial pathogens after using microbiome spray, and (iv) the change in the microbiome of the nose/throat region after using the microbiome spray.

Finally, some explorative (*post hoc*) analyses were included: (i) the relationship of the viral load to reported symptoms, (ii) colonization of the airways by the administered strains, and (iii) the correlation of the microbiome with several variables.

### Analysis of SARS-CoV-2 loads and SARS-CoV-2 IgG antibodies.

Combined nose-throat swabs were collected with eNAT swabs and stored at −20°C at the participants’ houses until all samples were collected at the end of the study (approximately 4 weeks after the start of the study). Samples for RNA extractions were transported to the Antwerp University Hospital (H. Goossens, Clinical Biology). RNA extractions were done with the EasyMAG kit with prior proteinase K treatment. The applied duplex real-time PCR used is based on methods described previously by Corman and colleagues (45) and on the detection of RNase P as described in the CDC protocol for real-time RT-PCR for the detection of the 2019 novel coronavirus (46). Both assays are combined in a real-time duplex assay running under the conditions of the former assay. The target gene for SARS-CoV-2 used in this study was the E gene, and RNase P was used as an internal control. When the cycle threshold (*C_T_*) value of RNase P was ≤35, the sample was of an acceptable quality. For SARS-CoV-2 IgG antibody analysis as a reflection of detectable antibody responses to SARS-CoV-2 in the placebo and treatment groups (see reference 25), blood fingerprick samples were stored on Whatman filter paper at −20°C or 4°C and analyzed via an in-house-developed Luminex bead-based assay targeting antibodies against the receptor-binding domain (RBD), the nucleocapsid protein (NCP), and spike proteins (S1S2) of the SARS-CoV-2 Wuhan strain (26, 47).

### Bacterial DNA extraction from combined nose-throat swabs and Illumina MiSeq 16S rRNA amplicon sequencing.

Self-collected combined nose-throat swabs were stored at −20°C at the participants’ houses until the end of the study. After transportation on ice, samples for DNA analysis were kept at −20°C in the laboratory until further processing. Prior to DNA extraction, all samples were vortexed for 15 to 30 s, and 500 μL of the eNAT buffer was used for automatic extraction using a DNeasy 96 PowerSoil Pro QIAcube HT kit (Qiagen). Negative extraction controls were included at regular time points throughout the study. All samples were eluted with 100 μL elution buffer, and DNA concentrations were measured using the Qubit 3.0 fluorometer (Life Technologies, Ledeberg, Belgium).

Amplicon sequencing (V4 region of the 16S rRNA gene) was performed using an in-house-optimized protocol (48, 49). Processing and quality control of the reads were performed using the R package DADA2, version 1.6.0, to achieve amplicon sequence variant (ASV)-level counts. Taxonomic annotation was performed using the ezbio 16S database (retrieved June 2018). All data handling and visualization were performed in R, version 3.4.4, using the tidyverse set of packages and the in-house package tidyamplicons, version 0.2.1 (publicly available at https://github.com/SWittouck/tidyamplicons), as described previously (48).

### Detection of administered *Lactobacillaceae* strains using qPCR.

Primers specific for *L. casei* AMBR2, *L. plantarum* WCFS1, and L. rhamnosus GG were used for qPCR (see reference 24). Primers 2759F (5′-CCCGGGCCGTTACGTTGCAGGCAAAA-3′) and 2841R (5′-ACTAGTTAATTGGTCAGTCGGTGCCC-3′) were used to target the *srr2* gene of *L. casei* AMBR2 (37), primers LGG_443_F (5′-CGTAGCTCTTTGCGTCATCT-3′) and LGG_443_R (5′-CGCATTGTATGCAGCCTTATTC-3′) were used to target the FM179322.1_443 locus of L. rhamnosus GG, and primers WCSF1_413_F (5′-GCCACAACACTTCAGCAATAC-3′) and WCSF1_413_R (5′-GTGCCATACACCCTGGTAAG-3′) were used to target the AL935263.2_413 locus of *L. plantarum* WCFS1. Four microliters of each extracted DNA sample was combined with 10 μL of Power SYBR green PCR master mix, 0.3 μL of each primer (20 μM), and 5.4 μL of RNase-free water. The *C_T_* value of each sample was used to calculate the concentration of the strain present in the sample. Nontemplate controls were included for each run.

### Sample size.

Studies that have tested the administration of beneficial lactobacilli into airways through a throat spray to exert respiratory effects and antiviral activity are very limited. Therefore, it was difficult to calculate the capacity for this type of potency study. Therefore, we looked for studies using other probiotic formulations on patients with COVID-19. Based on the available literature at the time when the study was designed, we aimed to recruit at least 150 individuals, 75 in each group. This sample size allows us to demonstrate a difference in the mean symptom score of 0.6 times the standard deviation (effect size of 0.6) between the two study groups (level of significance of 5%; power of 80%), taking into account a 10% dropout rate for each study group. However, recruitment was stopped earlier (*n* = 78) due to difficulties in finding sufficient numbers of study participants due to a drop in infection cases and the beginning of broad vaccination campaigns in Belgium.

### Statistical analyses.

A per-protocol analysis was performed on participants who completed the study and provided samples at all time points. Standardized scores (z scores) were used for the analysis of the primary outcome. The distributions of the severity scores for four different symptom summary scores on every day were compared between the treatment groups. Kaplan-Meier survival analysis was performed using the R survival package. The symptomatic tipping point was taken as the event, and the time until occurrence was tested in the different treatment groups. Differences between symptom scores in COVID-19-positive and -negative participants (based on PCR) were tested using a random-effects model, symptom ~ COVID-19 + (1|participant). *P* values were adjusted for multiple testing using Bonferroni’s correction. Differential bacterial abundances between treatment groups were tested with a random-effects model, CLR(ra) ~ treatment + plate + qubit_score + library_size + (1|participant), where CLR(ra) is the centered log ratio-transformed relative abundance of a given bacterium and plate, qubit_score, and library size constitute technical confounders. Effect sizes for the treatment group were calculated for time points *T*_2_ and *T*_3_, at which times the participants were using the spray. We corrected for multiple testing using the Benjamini-Hochberg procedure to control the false discovery rate (50).

### Data availability.

All data produced in the present study are available upon reasonable request to the authors. The sequencing data were deposited in the ENA under accession number PRJEB49183.
