# Supplementary material for: Randomized, Double-Blind, Placebo-Controlled Trial of a Throat Spray with Selected Lactobacilli in COVID-19 Outpatients
Source: Microbiol Spectr. 2022 Sep 26;10(5):e01682-22. doi: 10.1128/spectrum.01682-22 (PMC9604152; doi:10.1128/spectrum.01682-22)
Supplement: Supplemental file 1 — Fig. S1 to S3, Tables S1 to S4. Download spectrum.01682-22-s0001.pdf, PDF file, 0.6 MB [file spectrum.01682-22-s0001.pdf]

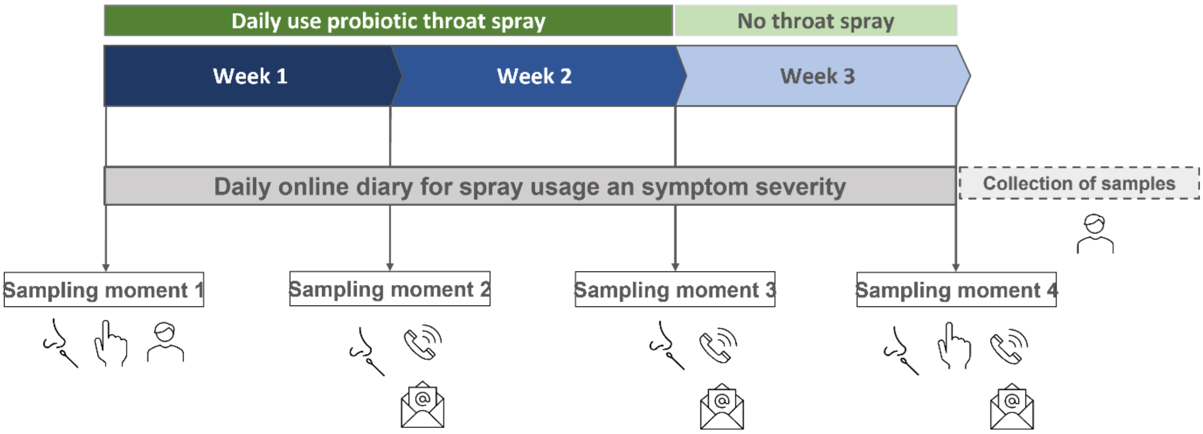

Figure S1: Overview of the study set-up. Patients were asked to use the verum spray or placebo for 14 days, with a recommended use of 5 times a day, which was also monitored via the online diary. After the intervention, patients were followed-up for an additional 7 days (so in total 21 days of symptom follow-up). They were asked to fill in an online diary via Qualtrics (Qualtrics, Provo, UT, USA) for each day of the study reporting spray usage as well as symptom severity. Every week, patients were contacted via teleconsults or email. At the end of the study, the study coordinator planned a final visit to collect all samples. Combined nose/throat swabs were collected at start (T1), after 1 week (T2), after 2 weeks (T3) and at the end of the trial (T4). Fingerprick blood samples were collected at T1 and T4.

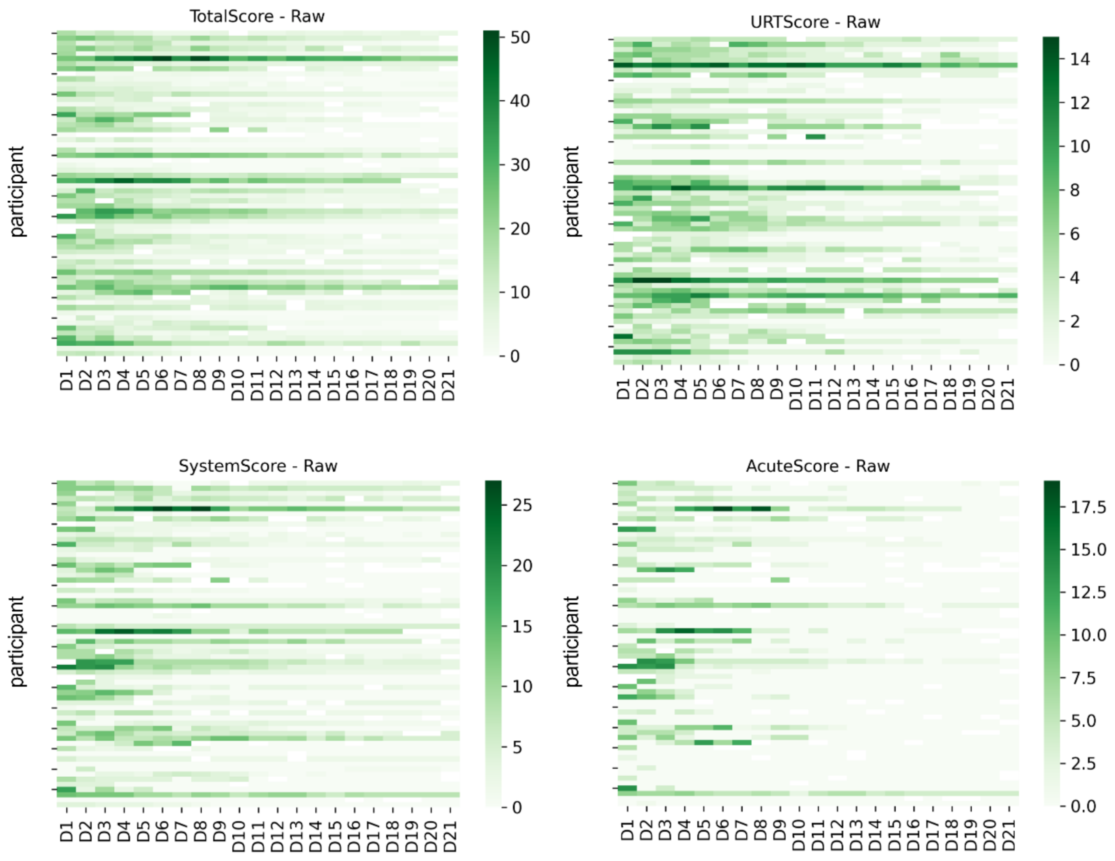

Figure S2: Raw symptom scores for the different scoring systems.

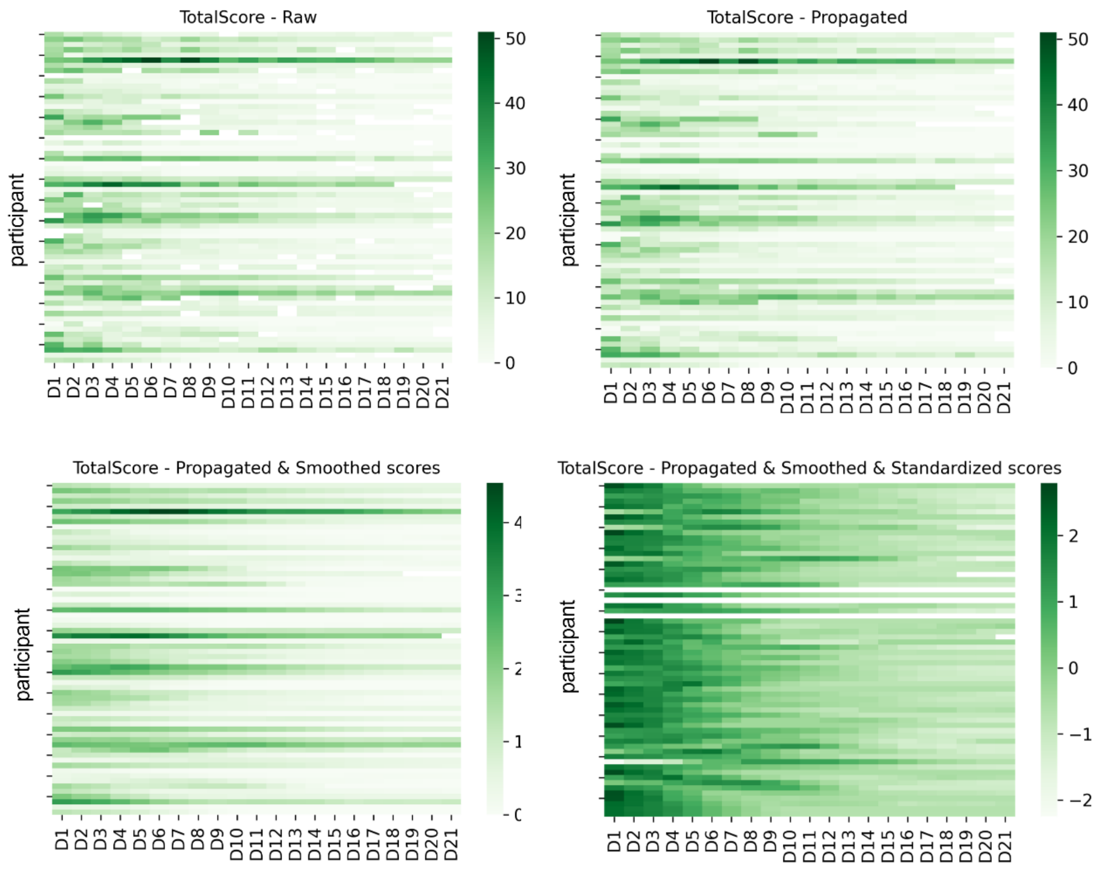

Figure S3: Example of symptom processing for the total score. Raw scores were first propagated, next smoothed and finally standardized (z-scores). The same analysis was done for the URT score, acute score and system score.

Table S1: Spray usage and absolute severity score in both study groups. Spray usage is depicted by the number of days the sprays were used (self-reported) and the remaining weight of the sprays (measured by the study team). Absolute severity scores (means ± stdev) for the different symptom summary scores are shown: total score (sum of all reported symptoms), local URT score (the sum of cough, sore throat, and nasal discomfort scores), acute score (the sum of fever, diarrhea, chills, and muscle pain scores), and system score (the sum of fever, shortness of breath, muscle pain, chills, fatigue, and diarrhea scores).

	**Verum (n = 33)**	**Placebo (n = 27)**
**Sprays used: number of days [mean, stdv]**	19 ± 3	19 ± 3
**Sprays used: remaining weight (grams)[mean, stdv]**	72.2 ± 5.4	71.7 ± 6
**Total score [mean, stdv]**		
• T1	13.4 ± 8.6	15.2 ± 9.3
• T2	10 ± 10.3	7.2 ± 6.9
• T3	6 ± 7	3.1 ± 4
• T4	4.3 ± 5.7	1.8 ± 3.1
**URT score [mean, stdv]**		
• T1	4.3 ± 3.4	5.3 ±3.2
• T2	3.3 ± 3.2	3.1 ± 2.7
• T3	2.3 ± 3	1.2 ± 2
• T4	1.5 ± 2.5	0.39 ± 0.6
**Acute score [mean, stdv]**		
• T1	3 ± 3	3.3 ± 3.5
• T2	1.4 ± 3.1	0.6 ± 1.3
• T3	0.3 ± 0.8	0.2 ± 0.7
• T4	0 ± 0	0.2 ± 0.5
**System score [mean, stdv]**		
• T1	6.1 ± 4.7	6.8 ± 5.4
• T2	4.4 ± 5.4	2.7 ± 3.2
• T3	2.3 ± 3.5	1.3 ± 1.9
• T4	1.6 ± 2.2	1 ± 1.7

Table S2: Correlation symptoms and being SARS-CoV-2 positive. A random effect model was used (symptom ~ covid + (1|person)).

Index	Estimate	Std. Error	Df	T value	Pr (>|t|)	Test	qvalue
covidTrue	0.314200	0.073	1260.49	4.29	1.96 e-05	Sore throat	2.94 e-04
covidTrue	0.428104	0.076	1268.74	5.63	2.15 e-08	Cold chills	3.22 e-07
covidTrue	0.476495	0.106	1259.19	4.47	8.18 e-06	Nasal cold	1.23 e-04
covidTrue	0.477897	0.085	1257.97	5.61	2.43 e-08	Fever	3.65 e-07
covidTrue	0.494028	0.090	1272.28	5.50	4.37 e-08	Cough	6.55 e-07
covidTrue	0.530083	0.103	1260.71	5.17	2.74 e-07	Muscle pain	4.11 e-06
covidTrue	0.578874	0.106	1257.84	5.44	6.43 e-08	Fatigue	9.64 e-07
covidTrue	0.822822	0.107	1261.76	7.69	3.08 e-14	Headache	4.63 e-13
covidTrue	1.266727	0.190	1258.04	6.67	3.76 e-11	URT score	5.63 e-10
covidTrue	1.494129	0.222	1264.11	6.73	2.53 e-11	Acute score	3.80 e-10
covidTrue	2.237859	0.298	1258.97	7.52	1.06 e-13	System score	1.59 e-12
covidTrue	4.420475	0.511	1258.08	8.64	1.70 e-17	Total score	2.60 e-16

Table S3: Mean relative abundances of *Lactobacillaceae* ASVs administered via the throat spray at the different timepoints in verum and placebo. A Wilcoxon test was used to evaluate statistical differences between verum and placebo group.

***Lactobacillaceae* ASV**	**Timepoint**	**Mean relative abundance in verum**	**Mean relative abundance in placebo**	**p-value**
L. casei ASV	T1	0.026905287	0.0001976367	0.30
	T2	0.025694896	0.0001236477	0.056
	T3	0.010757218	8.007239e-06	0.001
	T4	0.0001428949	2.530286e-05	0.37
L. plantarum ASV	T1	0.022253851	0.0002801401	0.53
	T2	0.019241153	0.0001108163	0.12
	T3	0.009505836	0.000000e+00	0.005
	T4	0.0001831164	8.910997e-05	0.24
L. rhamnosus ASV	T1	0.007690299	0.0003379468	1
	T2	0.007265016	0.0000000000	0.18
	T3	0.003396812	0.000000e+00	0.32
	T4	0.0000000000	5.718128e-06	0.006

Table S4: Average estimated CFU/ml counts for the applied *Lactobacillaceae* in verum and placebo based on qPCR and % of participants were the strains were detected. Of note, also in the placebo group we observed some detection, but this is probably because these lactobacilli are also present as endogenous members in low numbers in the respiratory tract.

**Strain**	**Estimated CFU/ml verum**	**Estimated CFU/ml placebo**	**% of participants detected verum**	**% of participants detected placebo**
L. casei AMBR2	1.1×10^8	9.1×10^3	85% (29/34)	23% (7/30)
L. rhamnosus GG	6×10^6	7.5×10^3	79% (27/34)	10% (3/30)
L. plantarum WCFS1	1.7×10^7	1.5×10^3	82% (28/34)	30% (9/30)

Table S5: Results of differential abundance tests. Statistical results from the differential abundance tests, including effect sizes, p-values and sizes.
